# Importance of Indigenous Breeds of Chicken for Rural Economy and Their Improvements for Higher Production Performance

**DOI:** 10.1155/2016/2604685

**Published:** 2016-04-07

**Authors:** Mahendra Kumar Padhi

**Affiliations:** Regional Centre, ICAR-Central Avian Research Institute, Baramunda, Bhubaneswar 751003, India

## Abstract

Indigenous/native breeds of chickens are playing an important role in rural economies in most of the developing and underdeveloped countries. They play a major role for the rural poor and marginalised section of the people with respect to their subsidiary income and also provide them with nutritious chicken egg and meat for their own consumption. Performance of native fowl can be improved by change in husbandry, feeding, and better health cover. However, genetic improvement may be made either through selection and crossbreeding or by utilisation of both selection and crossbreeding. Improvement through selection may be time consuming but the improvement will be permanent. Through crossbreeding improvement may be faster but research has to aim for the production of native-type birds with higher production potential. In the present review efforts have been made to present the importance of native fowl to rural economy and their improvement for higher production performance.

## 1. Introduction

Rural poultry farming using native breeds is being practised in many developing and underdeveloped countries throughout the world [1–6]. Importance of native birds for rural economy is immense in different countries [4, 7]. Though these birds are being used for rural backyard poultry production, their genetic potential has not been fully exploited. Improvements of native breeds through selection are being carried out, but still it has to be given more importance in different countries of the world [8–10]. Backyard farming has over the years contributed to a great extent to the agrarian economy of different countries. In the same way, rural backyard poultry production plays a vital role in the rapidly growing economy. It provides livelihood security to the family in addition to securing the availability of food. Unemployed youth and women can also earn an income through poultry farming. Indigenous breeds are well known for their tropical adaptability and disease resistance, while their plumage colour helps in protecting themselves against predators. The first priority of today's rural poultry farmer is not only having birds which lay just more eggs but also having birds which lay eggs with an optimum size as well as birds which grow to an optimum body weight with plumage colour similar to indigenous birds. Producers thus have a choice out of a number of native breeds. The present review was made to document the importance of indigenous chicken for rural economy and its upgradation/improvement with respect to performance.

## 2. Indigenous/Native Breeds of Chickens

Chickens are the most popular poultry worldwide irrespective of culture and region [11, 12]. Dessie et al. [11] reviewed the current state of knowledge on indigenous chicken genetic resources of the topics: domestication, distribution, and documentation of information on the genetic resources. Aini [13] reported the number of indigenous chickens in South East Asia. In India some of the important breeds/varieties which have been documented are Aseel, Ankaleshwar, Busra Chitagong, Daothigir, Denki, Ghagus, Haringhatta black, Kadaknath, Kalasthi, Kashmir Faverolla, Miri, Punjab Brown, Tellichery, Titri, Teni, Nicobari, Naked neck, and frizzle fowl [14, 15]. Besides this many nondescript desi chickens breeds are reported [16–18]. As per the report of Ramdas and Ghotge [19], there are approximately eight different strains or substrains of native chickens that are recognised by the communities of East Godavari district of Andhra Pradesh areas such as* Nati Kodi*,* Shankarjati kodi*,* Geesa kodi*,* Medajari Kodi*,* Rencha kodi*, or* Agees kodi*, and* Mattedu kodi*. Among these it is the Aseel that has been historically the breed of choice valued for its tasty meat, cockfighting abilities, agility, and ability to escape from predators.

Throughout the world indigenous/native breeds of chicken are reported. Adelake et al. [33] reported the performance of Nigerian local chicken which consist of normal feathers, frizzle, and Naked neck. Sola-Ojo and Ayorinde [31] documented the Fulani ecotype of Nigeria. Alewi et al. [32] reported the local* Kei* (a red plumage chicken) in Ethiopia. Reta [34] reported Horro, Tepi, and Jarso indigenous chickens in Ethiopia. Halima et al. [35] also reported the variation of native chicken population of North West Ethiopia. Mohammed et al. [36] documented the local chickens of Sudan such as large Baladi, bare neck, and Betwil. Like in India Bhuiyan et al. [37] reported number of native chicken breeds of Bangladesh like nondescript Deshi, Aseel, Naked neck, and Hilly. Venda chicken was seen in South Africa. Ovambo chicken originated from northern part of Namibia. Koekoek chickens are also found in South Africa. Naked neck trait known locally as Peel-neck chicken was reported by Mallia [38]. Aboe et al. [39] reported the productivity of free range village chickens on the Acra Plains of Ghana. Dorji et al. [40] reported the characterisation of Thai native chicken. Native chickens of Kenya reported in the literature are having different plumage colour [4, 41]. Vali [6] reported three indigenous chickens of Iran Naked neck, Marandi, and Public (compound of different groups). In China information is available on various indigenous chicken breeds like Xiaoshan, Xianju, Linghun, Bayiner, Wzgu, native sheak kai, YWC strain, Huiyang bearded chicken, Xinghua, Taihe silkies, Gushiu, Baijing fatty, Wenchung, and Quingyuan [42, 43].

Dessie et al. [11] documented the phenotypic characteristics of native chickens in tropics which include Matrouh, Mandarah, and Fayoumi breeds of Egypt and Tilili, Chefe, and Tepi of Ethiopia and southern, northern, and central ecotypes of Bolivia. They also reported the Ac and H'Mong of Vietnam, Kampung breed of Indonesia and Malaysia, Ching'wekwe, Kuchi, and Mbeya of Tanzania, local birds of Nigeria, Naked neck of Cameron, Koekoeak, Leowa-venda, Ovambo, and Naked neck of South Africa. They also provided a summarisation about Aseel, Kadaknath, Naked neck, and Sikkimese frizzle of India. The literature indicates that native fowl are more concentrated mostly in developing and underdeveloped countries than the developed countries.

## 3. Importance of Native Breeds for Rural Economy

Chickens in developing countries have more diverse use and benefits to household. The use of native chicken in tropics varies from region to region and from community to community within a region. In the tropics small land holders keep chickens for their socioreligious functions. This is because the commitment of an individual/community to a particular spiritual being, deity or season, and traditional and/or religious festivals is evaluated by the quality of the offering that satisfies special morphological features of the chicken demanded by the receiver [11]. Regardless of low output from native chicken in the tropics they can thrive and produce with irregular supply of feed and water and with minimum healthcare. They are part of balanced farming system and have vital roles in the rural households as a source of high quality animal protein and emergency cash income and play a significant role in the sociocultural life of the rural community. Though local chickens are slow grower and poor layers of small sized eggs they are, however, ideal mothers and good sitters [44], excellent foragers, and hardy [45] and possess natural immunity against common diseases [46, 47]. The small body size of native chickens is a desirable character in tropical and subtropical environment. One of the most important positive characters of native chickens is their hardiness, which is ability to tolerate the harsh environmental condition and poor husbandry practices (climate, handling, watering, and feeding) without much loss in production [47].

Nchinda et al. [48] reported that net margins from chicken husbandry represent 7.3, 3.2, and 2.2% of nonfood, food, and total monthly household expenditure, respectively, well above those of the not yet involved in family poultry in Haiti. The family poultry (chicken) husbandry support program was profitable for the beneficiary and contributed to the welfare of participants. Yang and Jiang [49] reported consumer preference for coloured feather and slow growing meat-type quality chickens in certain regions of the world. Quality chickens are generally produced by direct use of native chickens breed, which are generally slow growing with poor feed conversion. The sustained use of native chickens in the traditional or family poultry production system showed the need to consider the value of native chickens. Therefore, a stratified on farm analysis is required to apprise the needs and opportunities of the different production system for a realistic assessment of the economic value of different traditional traits [47]. Das et al. [50] reported that rural poultry production particularly chickens (followed by ducks production) play significant role in the socioeconomic development of Bangladesh. Almost 90% of all rural families keep a small number of native chickens and ducks under traditional free range semiscavenging systems. They reported that poultry are generally maintained by rural women and children that generate cash revenue and that supply adequate eggs and meat to their personal family's diet. Chickens generally scavenge around the homestead areas during day time, where they eat kitchen waste, left over cereal like rice, wheat, pulses, green grass, insects, and other available feed stuff. These waste feedstuffs are utilised by these native birds to produce a good quality, cheap source of animal protein. A study report on the small holder livestock development project (SLDP) in rural community at different rural areas of Bangladesh revealed that the overall socioeconomic conditions of the beneficiaries, their egg and meat consumption capability, empowerment of women in decision making issues, and employment opportunities significantly increased after intervention made by SLDP [51]. Because of these reasons that free range rural poultry keeping was deemed most suitable in Bangladesh as one of the developing countries, to provide rural women, landless poor, or marginal farmers with animal protein and earning for life [50].

Ramdas [52] reported that native women of East Godavari district, Andhra Pradesh, India, maintained over generations Aseel poultry and other local varieties. Birds managed under backyard system contribute crucially to women livelihood and are of critical cultural importance in the lives of native communities. More than 80% of the world poultry production is in village production system contributing up to 90% of poultry products in some developing countries [2]. Village poultry makes a substantial contribution to household food security throughout the developing world. It helps to diversify income, provides high quality food and fertilizer, and acts as form of household savings and insurance [53]. A study in the Niger delta showed that family poultry husbandry contributes 35% of the income of household's women and it is estimated at about 25% and 50% of Nigerian minimum wage and per capita income, respectively [54]. The very large numbers of native chicken breeds/ecotypes in the rural areas of most developing countries in Africa and Asia are due to their adaptation to village condition but also due to the preference given to meat and eggs produced by the local indigenous birds both in rural areas and in the cities. Although the meat and eggs produced by native birds are more expensive than those produced by commercial broilers or layers the latter are still beyond the purchasing power of the rural poor, who continue to rely on their own native birds for subsistence [2, 55].

Rural household's poultry production contributes 70% of the total production in most low income food-deficit countries [56]. Guèye [2] reported that indigenous chicken meat was 13% and 27% higher in market and supermarket compared to prices of meat from commercial chickens. Consumers with higher income group are willing to pay more in order to get indigenous meat. In Zambia Sayila [57] reported that indigenous chicken cost twice that of hybrid chicken. Egg prices were about 30% higher for traditional family based poultry production than the semi-industrial systems in North western Nicaragua [58]. Guèye [55] reviewed the employment and income generation through family poultry in low income food-deficit countries (LIFDC) and he presented details about field data relating to the contribution of family poultry to the household income in various LIFDC and these indigenous chickens were preferred. Bett et al. [1] published that attributes such as weight, body size, plumage colour and the general body condition significantly affect the indigenous price in Kenya. In Ethiopia birds endowed with red or white plumage colours combined with pea shaped comb types have always 15 to 35% exceed price values at marketing than those similar age birds do not having the above attributes [59]. Reta [34] reported that indigenous chicken of Ethiopia had a lot of conserved traits that fit to cultural, socio economical and environmental condition of the areas. They granted there owners with economic and nutritional benefits with no or little input supply in the village scavenging system. The importance and economic benefit from indigenous chicken are many. It is important to maximise the use of existing genetic diversity by improving current level of production in indigenous fowl [47, 60]. This will helps for sustainable use of existing genotypes that had adopted to the production environment in which they are maintained. In the recent past there is a growing concern to conserve biodiversity and to evaluate potential value of indigenous chicken not only for current but also for future unforeseen uses.

## 4. Up Gradation/Improvement of Indigenous Chickens for Higher Production

The diversity in agro-ecology, climatic conditions and variation in the purpose of chicken rearing in different regions and production environments in the tropics are believed to contribute to the current high diversity in chicken genetic resources in these areas. However, genetic improvements in the tropics on native indigenous chicken genetic resources are either rare or non-existent [47]. Instead in most instances developing countries uses high yielding commercial lines developed for intensified management system for crossbreeding with native fowl to increase the egg and meat production of native chicken by exploitation of heterocyst. Egypt has well developed breeds through long term crossbreeding and selection using local chicken population as foundation stocks [9].

Reports on native ecotypes in the tropics showed that their potential for egg production and growth is very low under smallholder farmer's management conditions. However, under improved feeding, housing and healthcare conditions, levels of production increased significantly [47]. The mean body weight gain of local chickens of Ethiopia under on station management was higher than traditional management [44, 60]. Abdelqader et al. [61] reported that there is significant improvement in performance of native fowl of Jordan with improving the management system alone. Hatchability, survivability, flock size, number of clutches, egg weight, and egg mass were the major parameters that improved significantly. Changes in traditional management practices can improve the performance of native chicken and thus contribute household incomes per year as reported in indigenous chicken in Bangladesh [62]. Supplemental feeding of hens during the incubation period was observed to be effective management tools in achieving a transition and from subsistence to economically viable semicommercial production. To be sustainable indigenous bird utilisation must efficiently meet current economic and social objectives without compromising the natural environment and recourses [53]. Changes in traditional management practices can improve the performance of native chicken and thus contribute household incomes per year as reported in native chicken in Bangladesh [62]. Okeno et al. [63] reported that utilisation of native chicken in their current genetic merit and production environment is more profitable under free range system and semi-intensive system but not economically viable under intensive system.

Moderate to high degree of heritability estimates (0.24 to 0.63) for juvenile traits and low to moderate estimates (0.14 to 0.33) in Nicobari fowl of India indicates the scope of improvements of this breed through selection [64, 65]. The magnitude of heritability estimates obtained in two Tanzanian chickens ecotypes indicates good prospects of improving different economic traits through selection [66]. In a selection programme in Horro chicken of Ethiopia the strong association between body weight at 16 weeks and egg production from 21 to 28 weeks and low to moderate heritability estimates for different traits indicates that the performance of Horro chicken can be improved through suitable selection programme [67]. An Iranian native population selected on the basis of breeding value recorded moderate to high heritability estimates and higher heritability estimates for body weight suggest improving the body weight and egg weight through selection and breeding programme can be achieved [68]. Haunshi et al. [69] reported moderate to high heritability estimates in Aseel (0.22 to 0.49) and Kadaknath (0.22 to 0.37) for juvenile body weight and shank length indicating there is scope for further improvement through selection.

There is potential for improvement of native chicken production. Improving the performance crossbreeding with Rhode Island Red, White Leghorns, Light Sussex, Black Australorp, and other synthetic breeds was initiated and reported by many authors [3, 4, 8, 21, 27, 70]. Upgrading the native chicken using high producing European breeds was seen as the quickest way of achieving genetic improvement, thus increasing egg and meat production [4]. Fulani ecotype of Nigeria and exotic egg-type chicken crossbreed was found to perform better than the native Fulani ecotype [31]. Crossbreed performs better than pure native chicken of Nigeria [33]. Alewi et al. [32] reported that local Kei performance could be improved by using the crossbreeds of Fayoumi and local Kei native chicken breeds. Effects of crossbreeding of exotic chicken with indigenous chicken reported in literatures with respect to different traits are presented in Tables 1, 2, 3, and 4. It was observed that except for few traits all the major economic traits improved in the crossbreeds compared to native chickens indicating that this is one of the tools to improve the performance of indigenous chickens. The dressing% and lean : bone ratio as well as n6 : n3 ratio was found to be better in indigenous chicken compared to crosses (Table 1). Body weight, egg weight, egg production, and age at sexual maturity were found to be better in crosses compared to indigenous chicken indicated in all of the studies reported using indigenous and exotic breeds (Tables 2, 3, and 4).

Breeding programme targeting improvement of indigenous chicken should focus on within breed selection rather than crossbreeding with commercial chicken breeds. This would help to maintain the indigenous chicken unique attributes which are appreciated by producers and avoid genetic erosion and dilution and contribute to their conservation [63]. Research in village poultry in different countries has revealed that the genetic potential of village chicken is generally not the major constraints to their production [71]. Iyer [10] was able to increase the annual egg production from 116 eggs to about 140 eggs per hen. The average egg weight of the flock also increased from 43 to 49 g through six generations of selection in a nondescript flock of Indian Desi fowl [3]. Menge et al. [72] suggested a bioeconomic model to support breeding of indigenous chicken in different production systems in Kenya. The breeding programme to improve the performance of indigenous breeds of chicken through selection is of great help to the farmers in the rural areas to improve their earing from indigenous birds. Though improvement through selection is slow, the change in production will be permanent in nature and maintain the unique characteristics of native/indigenous breeds.

## 5. Conclusions

The importance of native breeds of poultry birds for rural economy in developing and underdeveloped countries mostly in Asia and Africa is very high. They are part of balanced farming system that have vital roles in the rural households as a source of high quality animal protein and emergency cash income and play a significant role in the sociocultural life of the rural community and woman empowerment. One of the most important positive characters of native chicken is their hardiness, which is ability to tolerate the harsh environmental condition and poor husbandry practices without much loss in production. The native breed chickens are the reservoir of genomes and major genes for improvement of high yielding exotic germplasm for tropical adaptability and disease resistance. The low production performance of native breeds of chickens may be improved through improvement in husbandry practices, better healthcare, and supplementary feeds during lean season and also through selection and crossbreeding. Crossbreeding with exotic germplasm showed the improvement quickly; however, selection in native breeds can bring the improvement permanently. Upgradation of the native breeds of chickens through different breeding technique helps to increase the productivity of the germplasm and also their conservation in their natural habitat as the rural people will be very happy to rear them for their adoptability to harsh environment.

## Figures and Tables

**Table 1:** Effect of crossbreeding using indigenous birds with respect to feed conversion ratio, slaughter parameters, weight gain, and Heterophils/Lymphocytes (H/L) ratio.

Traits	Cross	Cross performance	Indigenous performance	Country	References
FCR	TI × (R × PR)	Better than indigenous	—	Thailand	[20]
	SB × BN	2.61	2.98	India	[21]
	SB × WN	3.52	3.12		
Slaughter wt.	TI × PR	1.48 kg	1.28 kg	Thailand	[22]
Dressing%	-do-	62.4	65.8	-do-	
Lean : bone ratio	-do-	1.09	1.23	-do-	
n6 : n3 fatty acid thigh	-do-	14.33	9.77	-do-	
Daily wt. gain of 4 wk	Thai indigenous cross	M = 25.24, F = 19.53	M = 19.52, F = 10.24	Thailand	[23]
H : L ratio	TI × (R × PR)	M = 0.37, F = 0.33	M = 0.32, F = 0.33	Thailand	[24]

F: female, M: male, R: Rhode Island Red, BN: Black Nicobari, PR: Plymouth rock, SB: synthetic broiler, TI: Thai indigenous, WN: White Nicobari, H : L: Heterophils : Lymphocytes, -do-: same as above, and n6 : n3: omega 6 to omega 3 fatty acid ratio.

**Table 2:** Effect of crossbreeding using indigenous birds with respect to body weight at 8 and 20 weeks of age.

Traits	Cross	Cross performance	Indigenous performance	Country	References
Body weight at 8 weeks (g)	DR × FU	M = 508, F = 468	M = 283, F = 252	Nigeria	[25]
	FU × DR	M = 390, F = 372			
	DR × Y	M = 429, F = 389			
	SB × BN	463	237	India	[21]
	SB × WN	444	252		
	BRN × WLH	M = 264, F = 229	M = 212, F = 195	India	[26]
	WLH × BRN	M = 269, F = 231			

Body weight at 20 weeks	DR × FU	M = 1360, F = 1275	M = 1191, F = 970	Nigeria	[25]
	FU × FR	M = 1333, F = 1333			
	DR × Y	M = 1336, F = 1143			
	SB × BN	1545	879	India	[27]
	SB × WN	1532	805	-do-	
	BRN × WLH	M = 868, F = 691	M = 709, F = 601	-do-	[26]
	WLH × BRN	M = 871, F = 692		-do-	
	K × J	M = 1587, F = 103	1079	-do-	[8]
	(K × J) × J	M = 2240, F = 1780		-do-	
	PB2 × A	998	896	-do-	[8]
	NP × DR	1414	1357	-do-	[8, 28]
	R × NU	1299	1382	-do-	[8, 28]
	NU × R	1304		-do-	
	PB2 × NU	2083		-do-	
	NU × R × R	1653		-do-	
	PB2 × NU × R	1878		-do-	
	DR × NJ	1058	878	-do-	[8]
	PB2 × NJ	1632		-do-	
	DR × (PB2 × NJ)	1525		-do-	
	(PB2 × NJ) × DR	1869		-do-	

A: indigenous fowl Guwahati, F: female, M: male, BN: Black Nicobari, BRN: Brown Nicobari, DR: Dahlem Red, FU: Fulani ecotype, K: Kadaknath, J: Jabalpur colour, NP: Palampur native, NJ: Ranchi native, NU: Udaipur native, PB2: meat-type synthetic breed, R: Rhode Island Red, SB: Synthetic broiler, WN: White Niocbari, WLH: White Leghorn, Y: Yoruba, and -do-: same as above.

**Table 3:** Effect of crossbreeding using indigenous birds with respect to body weight at 40 weeks and age at sexual maturity.

Traits	Cross	Cross performance	Indigenous performance	Country	References
Body weight at 40 weeks (g)	DR × FU	1537	997	Nigeria	[25]
	FU × DR	1320	-do-	-do-	
	DR × Y	1306	-do-	-do-	
	K × J	1761	1481	India	[8]
	(K × J) × J	1840		-do-	
	PB2 × A	2357	1366	-do-	[8]
	NP × DR	1738	1712	-do-	[8, 28]
	NP × RIR	1765		-do-	
	(NP × DR) × DR	1776		-do-	
	R × NU	1759	1502	-do-	[8, 28]
	NU × R	1680		-do-	
	PB2 × NU	2371		-do-	
	NU × R × R	1791		-do-	
	PB2 × NU × R	2250		-do-	

ASM (day)	Fayoumi × R	247.5	238.5	Bangladesh	[29]
	Aseel × DR	183	202	India	[30]
	DR × Aseel	181	202	-do-	
	SB × BN	163	197	India	[27]
	SB × WN	159	183	-do-	
	DR × Indigenous	157	159	Nigeria	[25]
	BRN × WLH	187	198	India	[26]
	WLH × BRN	170		-do-	
	Dominant Black × FU	145	158	Nigeria	[31]
	FU × Dominat Black	148		-do-	
	K × J	168	172	India	[8]
	(K × J) × J	163		-do-	

Annual egg production (number)	Aseel × DR	189	91	India	[30]
	DR × Aseel	191	91	-do-	-do-
	SB × BN	168	157	India	[27]
	SB × WN	178	162	-do-	
	BRN × WLH	186	144	India	[26]
	WLH × BRN	226		-do-	

A: indigenous fowl Guwahati, BN: Black Nicobari, BRN: Brown Nicobari, DR: Dahlem Red, FU: Fulani ecotype, K: Kadaknath, J: Jabalpur colour, NP: Palampur native, NU: Udaipur native, PB2: meat-type synthetic breed, R: Rhode Island Red, SB: synthetic broiler, WN: White Nicobari, WLH: White Leghorn, and Y: Yoruba.

**Table 4:** Effect of crossbreeding using indigenous birds with respect to egg weight and egg production up to 52 weeks of age.

Traits	Cross	Cross performance	Indigenous performance	Country	References
Egg weight (g)	SB × BN	55	48	India	[27]
	SB × WN	56	52	-do-	
	DR × indigenous	42.9	36.8	Nigeria	[25]
	BRN × WLH	47	45	India	[26]
	WLH × BRN	49		-do-	-do-
	Dominant Black × FU	51.45	47.19	Nigeria	[31]
	FU × Dominant Black	51.35		-do-	
	Local Kei × RIR	44.2	38.3	Ethiopia	[32]
	PB2 × A	40.56	36.49	India	[8]
	NP × DR	50.43	42.48	-do-	[8, 28]
	NP × RIR	49.56		-do-	
	(NP × DR) × DR	50.63		-do-	
	R × NU	50.32	48.40	-do-	[8, 28]
	NU × R	45.85		-do-	
	PB2 × NU	52.34		-do-	
	NU × R × R	53.42		-do-	
	PB2 × NU × R	53.84		-do-	

Egg production 52 weeks (no)	PB2 × A	70.23	66.30	-do-	[8]
	NP × DR	89.17	57.58	-do-	[8, 28]
	NP × R	87.44		-do-	
	R × NU	119.39	77.02	-do-	
	NU × R	102.90		-do-	
	PB2 × NU	96.23		-do-	
	NU × R × R	121.10		-do-	
	PB2 × NU × R	110.46		-do-	

A: indigenous fowl Guwahati, BN: Black Nicobari, BRN: Brown Nicobari, DR: Dahlem Red, FU: Fulani ecotype, NP: Palampur native, NU: Udaipur native, PB2: meat-type synthetic breed, R: Rhode Island Red, SB: synthetic broiler, WN: White Nicobari, WLH: White Leghorn, and -do-: same as above.
